# Consequences of COVID-19 Vaccine Hesitancy Among Healthcare Providers During the First 10 Months of Vaccine Availability: Scoping Review

**DOI:** 10.1177/08445621241251711

**Published:** 2024-05-02

**Authors:** Caitlyn D. Wilpstra, Sherry Morrell, Noeman A. Mirza, Jody L. Ralph

**Affiliations:** 1 Faculty of Nursing, 8637 University of Windsor, Windsor, Canada

**Keywords:** COVID-19, COVID-19 vaccines, health personnel, scoping review, vaccination, vaccination hesitancy

## Abstract

**Background:**

Throughout the COVID-19 pandemic, healthcare providers (HCPs)—including nurses—have played important roles in the vaccination effort. It is expected that COVID-19 vaccine hesitancy among HCPs has numerous consequences; however, the scope of these consequences and their impacts on providers, patients, and the broader healthcare system remained unclear.

**Purpose:**

To identify existing and emerging evidence to understand the state of knowledge of the consequences of COVID-19 vaccine hesitancy among HCPs.

**Methods:**

A scoping review was completed based upon the JBI scoping review methodology. The databases searched included OVID Medline, EBSCOhost CINAHL, ProQuest Nursing and Allied Health Source, ProQuest APA PsycInfo, and ProQuest Dissertations and Theses. The final literature search was completed on June 2, 2022. Studies were screened and retrieved based on predefined inclusion and exclusion criteria using Covidence reference management software. Data extraction followed criteria recommended in the JBI scoping review framework with additional relevant variables identified by the authors.

**Results:**

A total of 33 sources were included in the review. Consequences of HCP COVID-19 vaccine hesitancy were grouped under three themes and seven subthemes. Consequences affecting HCPs included health-related, psychosocial, and employment-related consequences. Consequences affecting patients pertained to COVID-19 vaccination communication and COVID-19 vaccination practices of HCPs. Consequences to the healthcare system involved consequences to coworkers and employment/attendance/staffing-related consequences.

**Conclusions:**

Healthcare provider COVID-19 vaccine hesitancy was found to have numerous consequences. By understanding the scope and extent of these consequences, healthcare leaders, researchers, and HCPs can work together to protect providers, patients, and healthcare systems.

## Background

Vaccination is a foundational component of global health (World Health Organization [WHO], 2022). Currently, the benefits of vaccination are seen in the role that COVID-19 vaccines have played in ameliorating the worst impacts of the COVID-19 pandemic. Between December 8, 2020, and December 8, 2021, an estimated 19.8 million deaths were prevented through availability of COVID-19 vaccines worldwide (Watson et al., 2022). In addition to mitigating COVID-related mortality, vaccines have helped to reduce COVID-19-related hospitalizations (Vilches et al., 2022) and the development of long-term symptoms following COVID-19 infection (Antonelli et al., 2022).

Vaccine hesitancy is a leading threat to global vaccine uptake (WHO, 2019). Vaccine hesitancy is often defined as the “delay in acceptance or refusal of vaccination despite availability of vaccination services” (MacDonald et al., 2015, p. 4163); however, the term was first used in 1994 regarding physicians who demonstrated reluctance to vaccinate their patients (Bedford et al., 2018). Research shows that vaccine hesitancy continues to be a pervasive phenomenon among healthcare providers (HCPs), including during the COVID-19 pandemic (Biswas et al., 2021; Browne et al., 2021; Li et al., 2023). In an international systematic review, Biswas et al. (2021) found that on average, 23% of HCPs demonstrated COVID-19 vaccine hesitancy, with rates ranging from 4.3%–72% of HCPs in individual studies. Furthermore, researchers have observed that nurses demonstrate higher rates of COVID-19 vaccine hesitancy than physicians (Browne et al., 2021; Li et al., 2023). This finding raises concerns as nurses are involved in many different aspects of immunization (International Council of Nurses, 2018) and often provide direct care to patients with COVID-19 (Gómez-Ochoa et al., 2021). During the first year of the pandemic, a global systematic review revealed that nurses suffered higher rates of COVID-19 infections than other HCPs (Gómez-Ochoa et al., 2021). While this study occurred prior to the development of COVID-19 vaccines, it is concerning that compared to other HCPs, nurses have been found to have both the highest rates of COVID-19 infections (Gómez-Ochoa et al., 2021) and some of the highest rates of COVID-19 vaccine hesitancy (Browne et al., 2021; Li et al., 2023).

Given the considerable rates of COVID-19 vaccine hesitancy among HCPs, it is necessary to understand the potential consequences of this phenomenon. While researchers have summarized the various determinants of HCP COVID-19 vaccine hesitancy (Biswas et al., 2021; Browne et al., 2021; Li et al., 2023), its consequences have not been identified to date in a scholarly review article. A recent narrative review on HCP vaccine hesitancy (excluding COVID-19 vaccines) found that vaccine-hesitant HCPs had lower personal vaccination rates, they were less likely to recommend vaccination to patients, and they were less committed to addressing vaccine hesitancy among patients (Verger et al., 2022). Unvaccinated HCPs have also suffered job losses when influenza vaccine mandates were implemented in healthcare organizations (Kitt et al., 2021). Therefore, while HCP COVID-19 vaccine hesitancy is expected to have similar consequences, the scope of these consequences and their impacts on providers, patients, and the broader healthcare system was unknown.

## Purpose

The purpose of this scoping review was to identify existing and emerging evidence in scholarly literature and academic theses in order to understand the state of knowledge of the consequences of COVID-19 vaccine hesitancy among HCPs. The review questions consisted of the following:

- What are the consequences of HCP COVID-19 vaccine hesitancy on HCPs themselves?
- What are the consequences of HCP COVID-19 vaccine hesitancy on patients?
- What are the consequences of HCP COVID-19 vaccine hesitancy on the healthcare system?

## Methods

### Design

A scoping review was performed based upon the methodology proposed in the JBI scoping review framework (Peters et al., 2020). In order to enhance the rigor of the review, the Preferred Reporting Items for Systematic Reviews and Meta-Analyses Extension for Scoping Reviews (PRISMA-ScR) was concurrently applied to the review process (Tricco et al., 2018).

### Inclusion criteria

#### Participants

Scholarly data pertaining to any member of the healthcare team working in an occupational role to provide direct or indirect healthcare to humans were considered for potential inclusion in this review. Articles on COVID-19 vaccine hesitancy exclusively among healthcare students and informal caregivers were excluded. An extremely broad definition of HCP was required for this review as many early pandemic studies provided poor conceptual and operational definitions of the term *healthcare provider,* and different titles and definitions were used for similar HCP roles in various countries.

#### Concept

The core concept in this scoping review was consequences associated with HCP COVID-19 vaccine hesitancy. *Consequences* were defined as any documented outcome, risk, or result of HCP vaccine hesitancy that affected the providers, their patients, or the healthcare system. Data describing consequences that exclusively affected the providers’ family or friends were excluded, as the focus of this review was consequences specific to the HCP role.

In scholarly literature, *vaccine hesitancy* has been defined as a behavior involving delayed or refused vaccination (MacDonald et al., 2015) and as an attitude of reluctance, concern, or fear surrounding vaccination (Bedford et al., 2018; Dubé et al., 2016). For the purpose of this review, *vaccine hesitancy* referred to both negative/hesitant vaccine attitudes or the HCPs’ own COVID-19 vaccine refusal/delay. Due to the emergent nature of the literature, non-vaccination in the presence of COVID-19 vaccine availability was considered to be a proxy for vaccine hesitancy. The HCPs’ vaccine hesitancy must have also logically preceded the documented consequences.

#### Context

Articles were limited to those involving COVID-19 vaccines. Studies on attitudes surrounding COVID-19 vaccines while they were in development were eligible for inclusion. Otherwise, the research context of the articles considered for inclusion was not limited.

### Sources of evidence

Potential sources of evidence included peer-reviewed quantitative, qualitative, or mixed-methods research articles, and scholarly editorials, opinion pieces, and other articles published in peer-reviewed journals. Academic theses and dissertations were also reviewed for inclusion. If a pertinent review article was identified, relevant primary sources were identified from the reference list and included in this review.

### Search methods

As per the JBI scoping review methodology, a three-step search process was followed (Peters et al., 2020). Brief, limited searches of OVID Medline and EBSCOhost CINAHL were completed in January 2022 to broadly assess the scope and availability of literature and to identify key terms and search headings (Peters et al., 2020). In the second step of the search process, key terms and search headings were synthesized into a repeatable search strategy that was conducted in all databases selected for use in this review (Peters et al., 2020). These databases included OVID Medline, EBSCOhost CINAHL, ProQuest Nursing and Allied Health Source, ProQuest APA PsycInfo, and ProQuest Dissertations and Theses. No time limits were placed on the search strategy. In the third phase of the search process, the reference lists of included sources were reviewed for additional relevant literature. The final literature search was completed on June 2, 2022. See Appendix A for an example of the search strategy used.

### Study selection

Once all literature searches were completed, the resultant citations were uploaded to Covidence (Veritas Health Innovation, Melbourne, Australia) for reference management. Articles in which exclusion criteria were immediately evident were excluded upon review of title or abstract. Two reviewers completed title and abstract reviews. Articles that appeared to meet inclusion criteria during abstract review were retrieved for full-text review. If the words “behavior” or “practice” were included in the title (referring to HCPs’ vaccination attitudes or hesitancy), these articles were retrieved for full-text review, as were any articles in which implications of HCP COVID-19 vaccine hesitancy on HCPs, patients, or the healthcare system were mentioned in the results, discussion, or conclusion of the abstract. Both reviewers completed full-text reviews, and all articles included in the review were agreed upon by both reviewers.

### Data extraction

The data extracted from each source consisted of that recommended in the JBI scoping review framework: the authors of each article and year of publication, study location, article objective, methodology, participant details and sample size, and findings (Peters et al., 2020). Additionally, data were gathered on the research design used, the specific healthcare setting involved, the dates of data collection, and how the concept of COVID-19 vaccine hesitancy/refusal was defined or quantified in each study. As suggested by JBI, the primary author tested the data analysis template on two relevant sources prior to evidence selection and data extraction (Peters et al., 2020).

### Data analysis and presentation

A complete table of all relevant data identified during data extraction was compiled. A narrative summary of the key findings of each included study was subsequently provided. The narrative summary was structured according to the three research questions: consequences of HCP COVID-19 vaccine hesitancy during the first 10 months of vaccine availability affecting HCPs themselves, consequences to patients, and consequences to the healthcare system. Within each of these three categories, data were further grouped by themes.

## Results

### Search results

**Figure 1.**
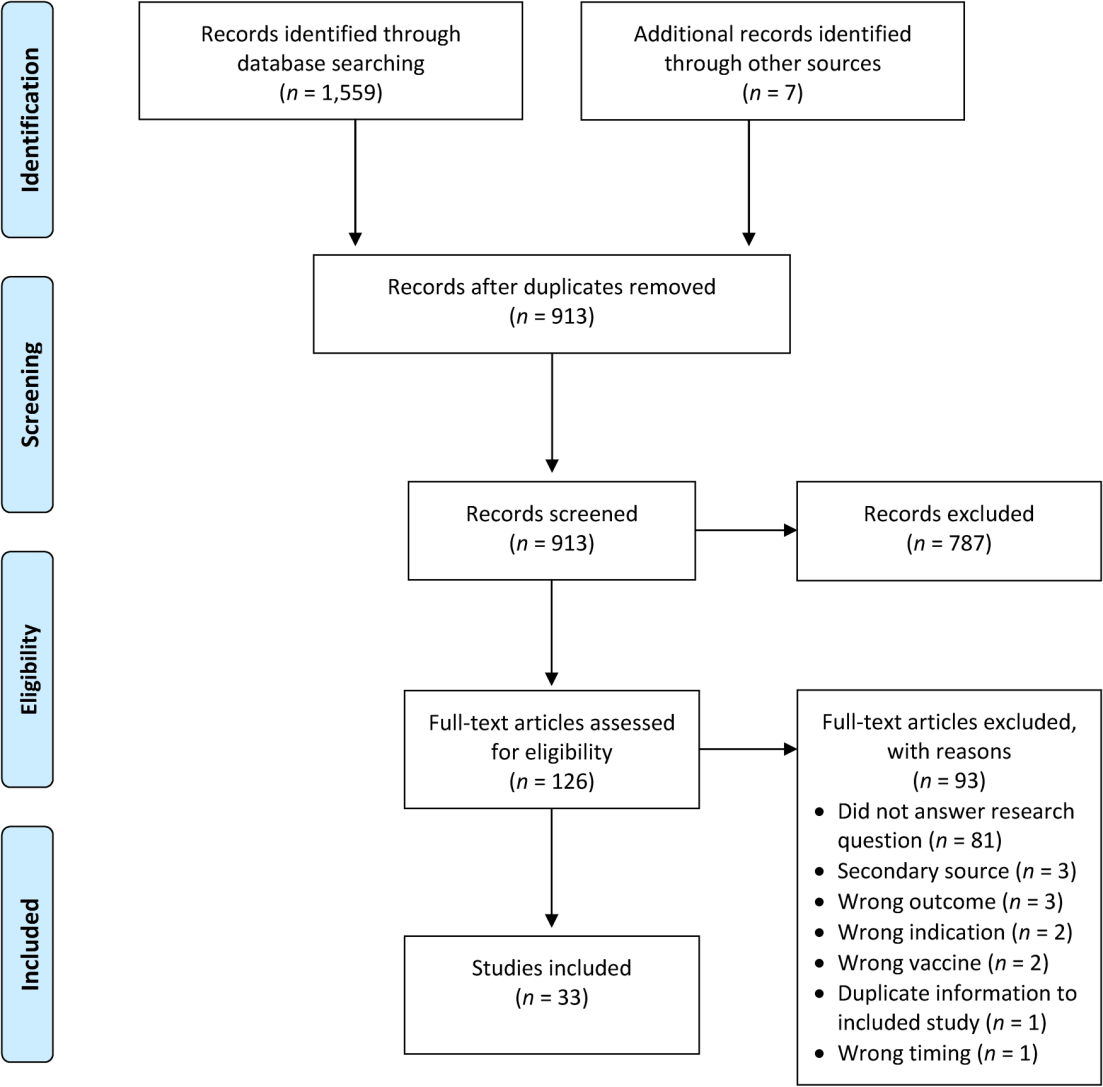
PRISMA flow diagram of search results. *Note:* Adapted from “Preferred Reporting Items for Systematic Reviews and Meta-Analyses: The PRISMA statement,” by Moher et al. (2009). *PLOS Medicine 6*(7), Article e1000097. Creative Commons Attribution License. https://doi.org/10.1371/journal.pmed.1000097.

A total of 1,559 references were retrieved from database searches. An additional seven articles were obtained from reference list searches for a total of 1,566 screened references. After 653 duplicate articles were removed, title and/or abstract reviews were completed for 913 articles, of which 787 studies were excluded based on pre-defined inclusion/exclusion criteria. Full-text articles were retrieved for 126 studies, and 93 of these studies were excluded for various reasons including not answering the research question (*n *= 81), not being a primary source (*n *= 3), wrong outcome (*n *= 3), wrong indication for the study (*n *= 2), wrong vaccine (*n *= 2), and wrong timing of data collection related to vaccine availability (*n *= 1). One editorial was excluded as it described the results of a study that was instead included in the review. Consequently, 33 articles were included in the scoping review. Of the additional seven studies included, six were identified from the reference lists of included studies, and one was retrieved from the previously described excluded editorial. See Figure 1 for a PRISMA flow diagram of the literature search results.

### Characteristics of included studies

All included studies were published between 2021–2022, with the data collection periods of these studies occurring during a 13-month period (September 2020–October 2021) that included the first 10 months of COVID-19 vaccine availability. The majority of studies (*n *= 20) were quantitative, and all of these utilized observational designs, including retrospective or prospective cohort studies (*n *= 12) and cross-sectional surveys (*n *= 8). Three studies utilized mixed-methods designs incorporating cross-sectional surveys and either semi-structured interviews or free-text qualitative questionnaires. Two studies were qualitative, consisting of a thematic analysis and a framework analysis. The remaining articles included editorials (*n *= 3), opinion pieces (*n *= 2), a quality assurance initiative (*n *= 1), a case study (*n *= 1), and a professional news article (*n *= 1).

The included evidence represents data from 14 countries including the USA (*n *= 7), the UK (*n *= 7), Greece (*n *= 3), Italy (*n *= 3), and Israel (*n *= 3). The remaining studies were conducted in Ireland (*n *= 1), France (*n *= 1), Germany (*n *= 1), Turkey (*n *= 1), India (*n *= 1), Hong Kong (*n *= 1), Thailand (*n *= 1), Japan (*n *= 1), and Australia (*n *= 1), and one location was unspecified. The study populations were highly varied, and different titles were used for similar HCP roles in various countries, which prevented aggregation of this data. However, in over half of the articles (*n *= 18), participants broadly defined as nurses or nursing personnel were included in the research samples or nursing exemplars were provided. In an additional five articles, the participants consisted of acute-care/hospital-based HCPs, which strongly suggests that nurses were included in these samples.

In most articles (*n *= 19), vaccine hesitancy was defined as COVID-19 vaccine refusal or non-vaccination. The authors of three articles studied HCPs’ lack of intention or unwillingness to receive COVID-19 vaccination. In two studies, vaccine hesitancy was construed as attitudes of concern, worry, doubt, or other negative attitudes toward COVID-19 vaccines. In the remaining nine studies, vaccine hesitancy was conceptualized as combinations of the preceding three categories or in alternate ways. See Table 1 for an abbreviated summary of all evidence included in the review.

**Table 1.** Abbreviated summary of evidence.

Author/Year	Objective	Methodology & Design	Study Location & Setting	Sample Size & Participants	Definition of Vaccine Hesitancy	Findings
Amit et al. (2021)	To determine COVID-19 rate reductions in healthcare providers (HCPs) following vaccination (BNT162b2 vaccine)	Quantitative: Retrospective cohort study	Israel: Hospital	9,109 HCPs eligible for COVID-19 vaccination	Unvaccinated HCPs	**Consequences to HCPs:** COVID-19 rates: Unvaccinated: 7.4/10,000 person-days. Vaccinated: Days 1–14: 5.5/10,000 person-days (adjusted rate reduction [aRR] 30%, 95% confidence interval [CI] 2–50). Vaccinated: Days 15–28: 3.0/10,000 person-days (aRR 75%, 95% CI 72–84). Symptomatic COVID-19 rates: Unvaccinated: 5.0/10,000 person-days. Vaccinated: Days 1–14: 2.8/10,000 person-days (aRR 47%, 95% CI 17–66). Vaccinated: Days 15–28: 1.2/10,000 person-days (aRR 85%, 95% CI 71–92).
Angel et al. (2021)	To assess the association between COVID-19 vaccination and symptomatic/ asymptomatic infection rates in HCPs (BNT162b2 vaccine)	Quantitative: Retrospective cohort study	Israel: Tertiary care hospital	6,710 HCPs and support staff (various roles; 1,701 participants employed in nursing roles included in sample)	Unvaccinated HCPs	**Consequences to HCPs:** Symptomatic COVID-19 rates: Unvaccinated: 149.8/100,000 person-days. Fully vaccinated: 4.7/100,000 person-days (adjusted incidence rate ratio [IRR] 0.03, 95% CI 0.01–0.06, *p *< .001). Asymptomatic COVID-19 rates: Unvaccinated: 67.0/100,000 person-days. Fully vaccinated: 11.3/100,000 person-days (adjusted IRR 0.14, 95% CI 0.07–0.31, *p *< .001).
Asaoka et al. (2022)	To compare symptoms of depression over time between HCPs willing and unwilling to accept COVID-19 vaccination	Quantitative: Cohort study (appears to be prospective)	Japan: Disaster care teams	597 disaster-team nurses, physicians, and other HCPs (71 nurses included in sample)	Willingness/ unwillingness to receive a COVID-19 vaccine	**Consequences to HCPs:** Standardized depressive (Patient Health Questionairre-9 [PHQ-9]) scores were statistically worse over time in vaccine-hesitant participants. PHQ-9 changes: Non-hesitant HCPs: 3.0 to 3.6. Vaccine-hesitant HCPs: 3.4 to 5.0 (Group/time interaction F[1, 207] = 3.9, *p *= .049).
Azamgarhi et al. (2021)	To determine COVID-19 vaccination rates and efficacy among HCPs (BNT162b2 vaccine)	Quantitative: Retrospective cohort study	UK: Tertiary care ortho-pedic hospital	2,235 HCPs and support staff (various roles; 494 nurses included in sample)	Unvaccinated HCPs	**Consequences to HCPs:** Time-based analysis of COVID-19 infections (≥ 14 days post-vaccination): Unvaccinated: 13/812 participants contracted COVID-19. Partially vaccinated: 7/1,392 participants contracted COVID-19 (Adjusted hazard ratio 0.30 [95% CI 0.09, 0.94], *p *= .04).
Bell et al. (2022)	To understand factors affecting COVID-19 vaccination in HCPs and social care workers	Mixed-methods: Cross-sectional survey and semi-structured interviews	UK: Settings not clearly specified	Quantitative: 1,656 HCPs and 261 social care workers (572 nurses/ midwives included in sample). Qualitative: 15 HCPs and 5 social care workers (roles unspecified).	Unvaccinated HCPs, as well as concerns and hesitant attitudes surrounding COVID-19 vaccination	**Consequences to HCPs:** Participants described loss of job security and threats of job loss. Unvaccinated participants reported breaches of normal medical privacy when management, rather than occupational health staff, contacted them about vaccination. Participants who were unvaccinated described an inability to discuss their concerns; they felt they were viewed as “anti-vaccination” and “stupid” (Bell et al., 2022, p. 19).
Bianchi, Germinario, et al. (2021)	To determine the short-term efficacy of the BNT162b2 COVID-19 vaccine among HCPs	Quantitative: Cohort study (appears to be retrospective)	Italy: Hospital	2,034 physicians and other unspecified HCPs	Refusal of COVID-19 vaccination	**Consequences to HCPs:** COVID-19 incidence rate: Unvaccinated: 2.45/1,000 person-days. Vaccinated: 0.54/1,000 person-days (IRR 0.22, 95% CI 0.15–0.32, *p *< .0001).
Bianchi, Tafuri, et al. (2021)	To determine the efficacy of the BNT162b2 COVID-19 vaccine among HCPs over a five-month period	Quantitative: Retrospective cohort study	Italy: Hospital	6,136 HCPs (various roles; 1,922 nurses included in sample)	Refusal of COVID-19 vaccination	**Consequences to HCPs:** COVID-19 incidence rate: Unvaccinated: 19.9/10,000 person-days. Vaccinated: 0.7/10,000 person-days (IRR 0.03, 95% CI 0.02–0.05, *p *< .0001). Symptomatic COVID-19 incidence rate: Unvaccinated:12.4/10,000 person-days. Vaccinated: 0.3/10,000 person-days (IRR 0.02, 95% CI 0.01–0.04, *p *< .0001). Symptom frequency: Unvaccinated HCPs with COVID-19 were more likely to experience six common symptoms of COVID-19 than vaccinated HCPs (*p *< .0001 for all symptoms). Hospitalization: Unvaccinated: 8 HCPs (1%) hospitalized. Vaccinated: 1 HCP (0.02%) hospitalized (*p *< .0001).
Can et al. (2022)	To determine COVID-19 vaccine efficacy during the alpha variant wave (CoronaVac vaccine)	Quantitative: Retrospective cohort study	Turkey: University hospital	3,174 HCPs and support staff (various roles; 726 nurses included in sample)	Unvaccinated HCPs	**Consequences to HCPs:** COVID-19 incidence rate: Unvaccinated: 133.7/100,000 person-days. Vaccinated: 70.7/100,000 person-days (IRR 0.53, 95% CI 0.41–0.69). Symptomatic COVID-19 incidence rate: Unvaccinated: 106.4/100,000 person-days. Vaccinated: 55.3/100,000 person-days (IRR 0.52, 95% CI 0.39–0.71).
Choi et al. (2022)	To assess HCPs’ confidence to accept and recommend COVID-19 vaccination, related to their role and ethnicity/race	Mixed-methods: Cross-sectional survey and semi-structured interviews	USA: Integrated health system	Quantitative: 2,948 HCPs (various roles; 1,184 nurses included in sample). Qualitative: 32 HCPs (various roles; 15 nurses included in sample).	Not consistently defined: refusal of vaccination and vaccine-hesitant attitudes	**Consequences to HCPs:** A nurse who refused vaccination contracted COVID-19 and described “a lot of remorse, a lot of regret” (Choi et al., 2022, p. 293). **Consequences to Patients:** A nurse who refused COVID-19 vaccination had such strong beliefs against vaccination that she refused to vaccinate patients against COVID-19.
Daskalakis et al. (2022)	To assess HCPs’ attitudes surrounding COVID-19 vaccination prior to and during pregnancy	Quantitative: Survey (appears to be cross-sectional)	Greece: Settings unspecified	1,226 HCPs (various roles; 76 nurses and 214 midwives included in sample)	Unvaccinated HCPs	**Consequences to Patients:** Unvaccinated HCPs were less likely to recommend COVID-19 vaccination during or prior to pregnancy (no quantitative data provided to support this statement).
Dennis et al. (2022)	To assess factors that promote or hinder COVID-19 vaccine acceptance among care home employees and their perceptions of mandatory vaccination	Qualitative: Semi-structured interviews	UK: Care homes	10 care home employees (unspecified roles)	Refusal of COVID-19 vaccination	**Consequences to HCPs:** Unvaccinated HCPs expressed intentions to leave their jobs when the vaccine mandate was introduced. An unvaccinated HCP described searching for a new job prior to the arrival of the mandate, leaving an intended long-term career. An unvaccinated HCP felt negatively judged by the general public. An unvaccinated HCP was forbidden by management from discussing the COVID-19 vaccine at work.
Deruelle et al. (2021)	To assess prenatal HCPs’ views on COVID-19 vaccination during pregnancy	Quantitative: Survey (appears to be cross-sectional)	France: University-affiliated hospitals, general hospitals, clinics, and other settings	1,416 Obstetricians and gynecol-ogists, general practitioners (GPs), and midwives	HCP willingness or unwillingness to accept a (future) COVID-19 vaccine	**Consequences to Patients:** HCPs who were unwilling to accept a future COVID-19 vaccine were less likely than non-hesitant HCPs to offer vaccination to pregnant women (adjusted odds ratio [OR] 5.52, 95% CI 2.14–14.24, *p *< .001).
Eschiti (2021)	A nurse's personal experience of vaccine hesitancy	Editorial: Self-described “case study”	USA: Setting unspecified	N/A: a case-study of the author's own vaccine hesitancy (1 nurse)	Self-described vaccine hesitancy/ doubts/concerns	**Consequences to HCPs:** The author described how nurses who have questions about the COVID-19 vaccine or different viewpoints risk ostracization and job loss.
Freckelton (2021)	To review a decision by the Irish High Court to suspend a physician's registration for promoting anti-scientific practices during the COVID-19 pandemic	Editorial: Review of an Irish High Court case	Ireland: Community	1 physician (GP)	Conscientious objection to COVID-19 vaccination	**Consequences to HCPs:** The physician's registration was suspended, in part, for failing to meet professional obligations related to his conscientious objection to COVID-19 vaccination. **Consequences to Patients:** The physician was unwilling to vaccinate patients against COVID-19. The physician did not properly inform his patients that he was a conscientious objector to vaccination or inform patients that other HCPs could administer COVID-19 vaccines to them instead.
Gesser-Edelsburg and Badarna Keywan (2022)	To assess physicians’ views on Pfizer COVID-19 vaccine knowledge acquisition, vaccine-hesitant coworkers, and vaccination communication with patients To assess the general public's views on physician knowledge of COVID-19 vaccination	Quantitative: Cross-sectional survey	Israel: Both hospital and community	295 physicians	Fears/ hesitancy/reservations surrounding COVID-19 vaccination	**Consequences to HCPs:** Participants indicated that the healthcare system has low tolerance for the views of vaccine-hesitant or vaccine-opposing physicians, as measured on an unspecified “tolerance index.”
Grace (2021)	To review the ethics of nurses sharing misinformation online	Ethics opinion/ review article	Location and setting unspecified	N/A: nursing examples provided	Delayed/refused COVID-19 vaccination and doubts about vaccine safety	**Consequences to Patients:** The author provided anecdotes of nurses sharing COVID-19 vaccine misinformation on social media and publicly sharing their decisions to delay or refuse COVID-19 vaccination.
Holzmann-Littig et al. (2021)	To assess German HCPs’ COVID-19 vaccine acceptance rates and determinants of vaccine hesitancy	Quantitative: Exploratory cross-sectional study	Germany: Hospital and community	4,500 HCPs and support staff (various roles; 466 nurses included in sample)	Undecided about/ unwilling to receive COVID-19 vaccine	**Consequences to Healthcare System:** When a majority of their (HCP) colleagues refused vaccination, participants were more likely to be vaccine hesitant (61.3% of participants whose colleagues refused vaccination were hesitant compared to 3.2% of participants whose colleagues accepted vaccination, *p *< .001).
Kaufman et al. (2022)	To understand the perceptions and learning needs of vaccine-hesitant individuals who were first prioritized for COVID-19 vaccination	Qualitative: Thematic analysis	Australia: Hospital, community/ private practice, and aged care settings	20 HCPs (various roles; 5 nurses included in sample) and 19 adults prioritized for COVID-19 vaccination	Doubts about receiving a COVID-19 vaccine and/or HCPs who were hesitant to recommend COVID-19 vaccination to patients	**Consequences to Patients:** Vaccine-hesitant HCPs stated that recommending COVID-19 vaccines fell outside of their scope of practice. Instead of speaking to the patients directly, they would suggest patients contact their primary care providers for COVID-19 vaccine information.
Kmietowicz (2021)	An article describing concerns surrounding the implementation of a COVID-19 vaccine mandate in the National Health Service (NHS)	Professional news article	UK: NHS	N/A	Unvaccinated HCPs	**Consequences to HCPs:** Upon implementation of a vaccine mandate, 126,000 HCPs in the NHS would leave their positions rather than get vaccinated (61% of the 208,000 total unvaccinated HCPs). **Consequences to Healthcare System:** Governmental concerns about HCP staffing shortages.
Lopez et al. (2022)	To determine rates of COVID-19 infections among vaccinated and unvaccinated HCPs during the Delta wave of the pandemic	Quantitative: Not specified (appears to be retrospective cohort)	USA: Veterans Affairs hospital and university-affiliated hospital	3,529 HCPs (unspecified roles)	Unvaccinated HCPs	**Consequences to HCPs:** Among all HCPs who received a COVID-19 test for suspected illness, the test positivity rates for unvaccinated HCPs at two hospital sites were 36.6% and 33.3% compared to 11.7% and 16.1% for vaccinated HCPs.
Maltezou et al. (2021)	To determine the impact of COVID-19 vaccination on HCP illness and workplace attendance (Pfizer-BioNTech vaccine)	Quantitative: Prospective cohort study	Greece: 5 tertiary care hospitals	7,445 HCPs and support staff (various roles; unspecified number of nursing personnel included in sample. Nursing personnel included nurses, midwifes, and nurse assistants)	Unvaccinated HCPs	**Consequences to HCPs:** COVID-19 incidence: Unvaccinated: 3.9% (*n *= 101). Vaccinated: 0.5% (*n *= 23, *p *< .001). Acute respiratory infection incidence: Unvaccinated: 1.7% (*n *= 44). Vaccinated: 0.5% (*n *= 23, *p *< .001). Influenza-like illness incidence: Unvaccinated: 2.5% (*n *= 65). Vaccinated: 0.7% (*n *= 33, *p *< .001). **Consequences to Healthcare System:** Rate of absenteeism after start of vaccination program: Unvaccinated: 11.8 episodes/100 HCPs. Vaccinated: 4.7 episodes/100 HCPs (*p *< .001). Mean duration of absence: Unvaccinated: 11.9 (standard deviation [SD] 8.1) days. Vaccinated: 6.9 (SD 5.7) days (*p *< .001). COVID-19-related work absence % among all absences (*n *= 535): Unvaccinated: 32.7%, (*n *= 101). Vaccinated: 10.2%, *n *= 23, *p *< .001). Absences related to COVID-19 exposure: Unvaccinated: 53.7%, *n *= 166). Vaccinated: 38.5%, (*n *= 87, *p *< .001). Mean duration of absence for COVID-19 exposure: Unvaccinated: 9.3 (SD 5.9) days. Vaccinated: 7.3 (SD 3.6) days, (*p *= .003). Mean duration of absence for fever: Unvaccinated: 13.9 (SD 7.3) days. Vaccinated: 2.2 (SD 2.9) days (*p *< .001). Mean duration of absence for influenza-like-illness: Unvaccinated: 19.9 (SD 10.8) days. Vaccinated: 7.5 (SD 7.6) days (*p *< .001).
Naleway et al. (2022)	To determine changes in COVID-19 vaccine efficacy among frontline workers during the Delta wave of the pandemic	Quantitative: Prospective cohort study	USA: Settings unspecified	4,549 frontline providers (various HCPs, first responders, and other essential workers; unspecified number of nurses included in sample; nursing data combined with allied health care providers’ data)	Unvaccinated HCPs	**Consequences to HCPs:** COVID-19 cumulative person/week incidence rate: Unvaccinated primary care providers: 4.7. Vaccinated primary care providers: 1.1. Unvaccinated nurses/allied health professionals: 5.9. Vaccinated nurses/allied health professionals: 1.1. Unvaccinated first responders: 10.6 Vaccinated first responders: 2.1.
Ntziora et al. (2022)	To determine the effects of HCP COVID-19 vaccine hesitancy on COVID-19 incidence and severity	Quantitative: Retrospective cohort study	Greece: 2 tertiary care hospitals	3,219 HCPs (various roles; 1,153 nurses included in sample)	Unvaccinated HCPs	**Consequences to HCPs:** Non-vaccination was significantly associated with contracting COVID-19 (OR 11.54, 95% CI 10.75–12.40, *p *< .001). COVID-19 incidence: Unvaccinated: 29.5% (*n *= 88). Vaccinated: 3.5% (*n *= 102). COVID-19 hospitalizations: Unvaccinated: 4.5% (*n *= 4). Vaccinated: None. COVID-19 deaths: Unvaccinated: 1.1% (*n *= 1). Vaccinated: None.
Papini et al. (2022)	To examine COVID-19 vaccine attitudes, behaviours, and information sources among Italian HCPs	Quantitative: Cross-sectional survey	Italy: Hospital and community	2,137 HCPs and support staff (various roles; 894 nurses and 100 auxiliary nurses included in sample)	Providing a nonmedical reason for COVID-19 vaccine refusal or refusing to recommend the vaccine	**Consequences to Patients:** Vaccine-hesitant HCPs were less likely to recommend COVID-19 vaccination to patients than non-hesitant HCPs (OR 12.90, 95% CI 7.26–69.23, *p *= .00 [sic]).
Poon et al. (2021)	To understand what factors affect family physicians’ likelihood to recommend the COVID-19 vaccine to patients	Quantitative: Cross-sectional survey	Hong Kong: Public and private practice	312 family physicians/ GPs	Unvaccinated HCPs	**Consequences to Patients:** The 3 physicians who refused vaccination would neither recommend COVID-19 vaccination to patients nor initiate patient vaccination discussions. 24/28 physicians (85.7%) considering vaccination within the next year would also not recommend vaccination to patients, and 67.9% (*n *= 19) would not initiate vaccination discussions. Vaccinated physicians were significantly more likely to recommend COVID-19 vaccines to patients than unvaccinated physicians (adjusted OR 12.23, 95% CI 3.45–43.33, *p *< .001).
Poyiadji et al. (2022)	To review the potential clinical implications of a mandatory COVID-19 vaccination policy on a radiology department and strategies to ameliorate these impacts	Quality assurance initiative	USA: Level I, II, and III trauma center hospitals	1,506 radiology department employees (unspecified number of nurses included in sample)	Remaining unvaccinated in the presence of a COVID-19 vaccine mandate	**Consequences to HCPs:** 400 employees in the hospital network refused COVID-19 vaccination and resigned, including 14 radiology employees who retired/resigned (0.9% of department). **Consequences to Healthcare System:** 1 hospital site was forced to close their outpatient MRI department for 1 day due to inadequate staffing. For 4 weeks prior to the final deadline for vaccination, the number of MRIs performed at one hospital site fell 1.9%–5.8%.
Ravindra Naik et al. (2022)	To assess differences in chest CT severity scores between vaccinated and unvaccinated HCPs who tested positive for COVID-19	Quantitative: Cross-sectional survey	India: Tertiary care center	2,043 HCPs (unspecified roles)	Unvaccinated HCPs	**Consequences to HCPs:** Chi square analysis demonstrated significant differences in chest CT severity scores between the vaccinated and unvaccinated participants (*p *< .001): Normal chest CT severity score: Unvaccinated: 19.07% (*n *= 197). Vaccinated: 50.89% (*n *= 514). Mild chest CT severity score: Unvaccinated: 40.27% (*n *= 416). Vaccinated: 37.82% (*n *= 382). Moderate chest CT severity score: Unvaccinated: 25.75% (*n *= 266). Vaccinated: 10.99% (*n *= 111). Severe chest CT severity score: Unvaccinated: 14.91% (*n *= 154). Vaccinated: 0.3% (*n *= 3).
Redmond et al. (2022)	To compare COVID-19 incidence and symptoms between vaccinated and unvaccinated HCPs	Quantitative: Not specified (appears to be retrospective cohort)	USA: Veterans Affairs medical center	5,630 HCPs (unspecified roles)	Unvaccinated HCPs	**Consequences to HCPs:** COVID-19 cumulative incidence: Unvaccinated: 5.7% (*n *= 80). Vaccinated: 0.3% (*n *= 12, *p *< .0001). **Consequences to Healthcare System:** 4 clusters of COVID-19 cases among 17 staff members were most likely transmitted through unvaccinated employees; no outbreaks were linked to vaccinated staff. An unvaccinated employee transmitted COVID-19 to a vaccinated colleague. Several unvaccinated HCPs were required to quarantine from work after COVID-19 exposures.
Ritter et al. (2021)	To review the process of promoting COVID-19 vaccine uptake among nursing home staff prior to implementing a vaccine mandate	Case study	USA: Skilled nursing home	246 nursing home employees (unspecified roles)	Remaining unvaccinated in the presence of a COVID-19 vaccine mandate	**Consequences to HCPs:** 17 employees resigned (6.9% of all employees). **Consequences to Healthcare System:** Loss of 17 employees.
Savic et al. (2022)	To describe the debate surrounding mandatory vaccination of NHS staff in the UK and the impact of HCPs who spoke out against mandatory vaccination	Editorial	UK: Setting N/A	N/A: the editor discussed the case of a single vaccine-hesitant physician	Non-vaccination in the presence of a COVID-19 vaccine mandate and doubts surrounding vaccination	**Consequences to Patients:** After an intensive care unit physician spoke out against COVID-19 vaccination, the editor described a “negative impact… on the public discourse around vaccination” (Savic et al., 2022, p. 608).
Sirikalyanpaiboon et al. (2021)	To assess physicians’ attitudes toward and acceptance of COVID-19 vaccines as well as predictors of vaccine acceptance	Quantitative: Cross-sectional survey	Thailand: University-affiliated teaching hospital	705 physicians (staff, residents, and fellows)	Uncertain about or unwilling to accept COVID-19 vaccination	**Consequences to Patients:** 64.5% of vaccine-hesitant physicians were unwilling or hesitant to recommend COVID-19 vaccination to patients, compared to 16.6% of non-hesitant physicians.
Sokol (2021)	An ethicist argues for mandatory COVID-19 vaccination among HCPs	Opinion piece, including a small amount of quantitative data to support arguments	UK: Setting unspecified	N/A	Refusal of COVID-19 vaccination	**Consequences to Healthcare System:** 90% of NHS trust leaders worried that mandatory vaccination (and loss of employees) would lead to staffing shortages in both health and social care services.
Woolf et al. (2022)	To assess HCPs’ perspectives on mandatory COVID-19 vaccination	Mixed-methods: Cross-sectional survey and free-text, qualitative questionnaire	UK: Settings unspecified	3,235 HCPs and support staff (various roles; 698 nursing staff included in sample; nursing staff also included nurse assistants and midwives)	As described in a prior study (Woolf et al., 2021): Uncertainty about/unwillingness to accept a future or actual COVID-19 vaccine	**Consequences to Patients:** Vaccine-hesitant HCPs were significantly less likely than non-hesitant HCPs to support mandatory COVID-19 vaccination for the general public and HCPs (OR 0.49, 95% CI 0.39−0.63, *p *< .001).

### Review findings

#### Consequences to healthcare providers

Consequences of HCP COVID-19 vaccine hesitancy affecting the HCPs themselves were described in 22 articles. These consequences fell under the themes of health-related, employment-related, and psychosocial consequences.

**Health-Related Consequences.** Vaccine-hesitant HCPs were found to experience numerous consequences that affected their physical health. Unvaccinated HCPs working in hospitals (Amit et al., 2021; Angel et al., 2021; Azamgarhi et al., 2021; Bianchi, Germinario, et al., 2021; Bianchi, Tafuri, et al., 2021; Can et al., 2022; Maltezou et al., 2021; Naleway et al., 2022; Ntziora et al., 2022; Redmond et al., 2022) and community settings (Naleway et al., 2022) both experienced higher rates of COVID-19 than vaccinated HCPs. Unvaccinated HCPs also experienced higher rates of symptomatic (Amit et al., 2021; Angel et al., 2021; Bianchi, Tafuri, et al., 2021; Can et al., 2022) and asymptomatic COVID-19 infections (Angel et al., 2021). Among a sample of HCPs with COVID-19 symptoms, unvaccinated participants had polymerase chain reaction test positivity rates two to three times higher than vaccinated participants (Lopez et al., 2022). Unvaccinated HCPs in Maltezou et al.'s (2021) study also had higher rates of non-COVID-related illnesses than vaccinated participants, including influenza-like and acute respiratory illnesses.

Additionally, unvaccinated HCPs were found to experience more severe complications of COVID-19 infections than vaccinated providers. Among a large sample of HCPs in India with confirmed COVID-19 infections, chi-square analysis revealed that unvaccinated participants had worse chest computerized tomography severity scores than vaccinated participants (*p *< .001) (Ravindra Naik et al., 2022). For example, 14.91% (*n *= 154) of unvaccinated HCPs had severe computerized tomography severity scores, compared to 0.3% (*n *= 3) of vaccinated HCPs (Ravindra Naik et al., 2022). In a study of HCPs at an Italian hospital, 1% (*n *= 8) of unvaccinated participants were hospitalized with COVID-19 compared to 0.2% (*n *= 1) of vaccinated participants (*p *< .0001) (Bianchi, Tafuri, et al., 2021). Likewise, among a sample of hospital-based HCPs in Greece, 4.5% (*n *= 4) of unvaccinated participants with COVID-19 required hospitalization, and one HCP died (Ntziora et al., 2022). No vaccinated participants experienced these outcomes (Ntziora et al., 2022).

**Psychosocial Consequences.** Five articles described psychosocial consequences of HCP COVID-19 vaccine hesitancy. Vaccine-hesitant health and social care workers in the UK qualitatively “felt they were dismissed as being anti-vaccination and treated as stupid” (Bell et al., 2022, p. 19). Similarly, Eschiti (2021) described nurses’ concerns of ostracization for having doubts about COVID-19 vaccination, while care home employees in the UK felt judged by the general public for not receiving the vaccine (Dennis et al., 2022). One nurse in the USA experienced “a lot of remorse, a lot of regret” (Choi et al., 2022, p. 293) after refusing vaccination and subsequently contracting COVID-19. Quantitatively, COVID-19 vaccine hesitancy was associated with worse depression scores over time among Japanese disaster medical and psychiatric team members, with average depression scores rising from 3.4 to 5.0 in hesitant participants compared to 3.0 to 3.6 in non-hesitant participants (F[1, 207] = 3.9, *p *= .049) (Asaoka et al., 2022).

**Employment-Related Consequences.** Eight articles summarized the employment-related consequences encountered by vaccine-hesitant HCPs, which often occurred when COVID-19 vaccine mandates were implemented. In the UK, 126,000 HCPs working in the National Health Service (NHS) indicated they would leave their jobs, rather than get vaccinated upon implementation of a COVID-19 vaccine mandate (Kmietowicz, 2021)—a finding that was paralleled in Dennis et al.'s (2022) qualitative study of care home providers in the UK. Poyiadji et al. (2022) and Ritter et al. (2021) described how 400 unvaccinated hospital-network employees and 17 unvaccinated skilled nursing home employees, respectively, were forced to resign or retire when COVID-19 vaccine mandates were implemented. In other articles, vaccine-hesitant HCPs described a loss of job security (Bell et al., 2022; Eschiti, 2021) or an unexpected change in career plans (Dennis et al., 2022). In a single case study, the Irish High Court suspended a physician's professional registration for reasons which included his failure to meet professional obligations while conscientiously objecting to COVID-19 vaccination (Freckelton, 2021).

Healthcare providers in the UK also qualitatively reported breaches in medical privacy at their workplaces when COVID-19 vaccination compliance was overseen by management rather than occupational health departments (Bell et al., 2022). In another study, management prohibited an unvaccinated care home employee from discussing the COVID-19 vaccine with colleagues, for fear of this employee's influence on others (Dennis et al., 2022). Physicians in Israel similarly indicated that the healthcare system as a whole had low tolerance for allowing vaccine-hesitant or vaccine-opposing physicians to critique or express doubts about the COVID-19 vaccine (Gesser-Edelsburg & Badarna Keywan, 2022).

#### Consequences to patients

The patient-related consequences of HCP COVID-19 vaccine hesitancy were outlined in 11 articles. The effects on patients fell under two themes: consequences affecting COVID-19 vaccine communication and consequences related to COVID-19 vaccination practices of HCPs.

**Consequences Related to COVID-19 Vaccination Communication.** Six articles outlined the consequences of HCP COVID-19 vaccine hesitancy on patient vaccine recommendations. In a qualitative study of Australian HCPs, vaccine-hesitant participants believed that recommending COVID-19 vaccines was beyond their scope of practice (Kaufman et al., 2022). Unvaccinated physicians in Hong Kong similarly avoided initiating COVID-19 vaccine discussions with patients (Poon et al., 2021). Among a sample of HCPs in Thailand, 64.5% of hesitant providers were not willing to recommend vaccination to patients compared to 16.7% of non-hesitant providers (Sirikalyanpaiboon et al., 2021). Two studies identified, with statistically significant odds ratios, that vaccinated physicians were more likely to recommend (Poon et al., 2021) and unvaccinated HCPs were more likely to not recommend COVID-19 vaccination to patients (Papini et al., 2022). An additional two studies found that obstetrical care providers who were unvaccinated (Daskalakis et al., 2022) or unwilling to receive a COVID-19 vaccine (Deruelle et al., 2021) were less likely than willing/vaccinated HCPs to recommend COVID-19 vaccination during (Daskalakis et al., 2022; Deruelle et al., 2021) or prior to pregnancy (Daskalakis et al., 2022).

One editorial revealed an alternate communication platform where vaccine-hesitant HCPs expressed their opinions. Grace (2021) provided examples of nurses sharing reasons for delaying or refusing COVID-19 vaccines on social media. Other nurses promoted COVID-19 vaccine misinformation on social media, which was noted to be particularly unethical when nurses’ credentials were used (Grace, 2021). Savic et al. (2022) similarly described how the general public's vaccination discourse was negatively affected when a video clip of an unvaccinated intensivist in the UK speaking out against COVID-19 vaccines was widely shared on social media.

**Consequences Related to COVID-19 Vaccination Practices of Healthcare Providers.** The effects of HCP COVID-19 vaccine hesitancy on vaccination practices affecting patients were outlined in three articles. As described in a mixed-methods study (Choi et al., 2022) and an editorial (Freckelton, 2021), an American nurse and an Irish physician, respectively, refused to vaccinate patients against COVID-19. The physician described by Freckelton (2021) also declined to refer patients to another HCP for vaccination. Finally, vaccine-hesitant HCPs working in various roles were nearly 50% less likely than non-hesitant HCPs to support COVID-19 vaccine mandates for both HCPs and the general public (Woolf et al., 2022).

#### Consequences to the healthcare system

The consequences of HCP COVID-19 vaccine hesitancy also affected the healthcare systems where these HCPs worked. These consequences were detailed in seven articles. The repercussions to the healthcare system fell under two general themes: consequences to coworkers, and consequences affecting employment, attendance, and staffing.

**Consequences to Coworkers.** Unvaccinated HCPs precipitated workplace COVID-19 transmission between 17 staff members of a Veterans Affairs medical center in the USA (Redmond et al., 2022). In one instance, transmission occurred from an unvaccinated HCP to a vaccinated colleague (Redmond et al., 2022). HCPs’ hesitancy was also statistically associated with their coworkers’ likelihood to receive COVID-19 vaccination (Holzmann-Littig et al., 2021). In a sample of German HCPs, participants were more likely to be hesitant toward the COVID-19 vaccine if they reported that their coworkers refused vaccination (61.3% of participants hesitant) compared to those who reported that their coworkers accepted vaccination (3.2% of participants hesitant, *p *< .001) (Holzmann-Littig et al., 2021).

**Employment/Attendance/Staffing-Related Consequences.** COVID-19 vaccine hesitancy among HCPs was linked with numerous impacts on healthcare organizations’ abilities to staff and carry out clinical services. A UK governmental (House of Lords) committee expressed serious concerns about staff shortages upon implementation of an NHS vaccine mandate (Kmietowicz, 2021)—a worry also shared by 90% of NHS trust leaders in the UK (Sokol, 2021). In two studies, a hospital network (Poyiadji et al., 2022) and a skilled nursing facility (Ritter et al., 2021) lost 400 unvaccinated HCPs and 17 unvaccinated HCPs, respectively, to COVID-19 vaccine mandates. Within the hospital network's radiology department, the loss of 14 employees contributed to 1.9%–5.8% fewer MRIs performed in the weeks preceding the mandate and the closure of an MRI department for one day upon implementation of the mandate (Poyiadji et al., 2022).

In a study of five acute-care hospitals in Greece, hospitals encountered higher rates and longer durations of staff absences among unvaccinated HCPs (Maltezou et al., 2021). Significantly more absences were related to COVID-19 illness in unvaccinated HCPs (32.7%) than in vaccinated HCPs (10.2%, *p *< .001) (Maltezou et al., 2021). Finally, two studies addressed the loss of unvaccinated HCPs to mandatory COVID-19 quarantine periods (Maltezou et al., 2021; Redmond et al., 2022). In Maltezou et al.'s (2021) study, mandatory quarantine periods were a reason for 53.7% of absences in unvaccinated participants compared to 38.5% of absences in vaccinated participants (*p *< .001). Unvaccinated HCPs also remained off work longer than vaccinated providers when they were exposed to COVID-19 (Maltezou et al., 2021).

## Discussion

Among the 33 articles included in this review, numerous consequences of HCP COVID-19 vaccine hesitancy were identified, affecting the HCPs themselves, their patients, and the broader healthcare system. When interpreting the results, it must be noted that all articles represent data collected prior to the arrival of the first SARS-CoV-2 Omicron variant, which was deemed a variant of concern on November 26, 2021 (WHO, 2021). This timeline is significant for two important reasons. First, compared to earlier COVID-19 variants, the Omicron variant has been found to cause less severe illness and lower hospitalization rates than the earlier Alpha and Delta variants (Christensen et al., 2022). Second, COVID-19 vaccine efficacy against the Omicron variants is notably lower and of shorter duration compared to earlier variants of the virus (Andrews et al., 2022; Christensen et al., 2022). Therefore, this review reflects a period of the pandemic when vaccination provided good immunity against more severe strains of the virus.

Nevertheless, these existing and emergent studies describe what is known about the consequences of HCP COVID-19 vaccine hesitancy during the first 10 months when vaccines were made available to HCPs. This timeframe is important to examine as the newness of a vaccine contributes toward vaccine hesitancy (MacDonald et al., 2015), and HCPs were one of the first groups offered the novel COVID-19 vaccines (Angel et al., 2021). While some of the documented consequences may have evolved with subsequent variants of the virus, the review findings describe the repercussions that occurred when members of occupations at high-risk for COVID-19 exposures (Gómez-Ochoa et al., 2021), trusted by the public, and essential for the function of the healthcare system refused or hesitated to receive a new vaccine amidst a global pandemic.

The most frequently described consequence of HCP vaccine hesitancy was higher rates of COVID-19 infections in unvaccinated HCPs (Amit et al., 2021; Angel et al., 2021; Azamgarhi et al., 2021; Bianchi, Germinario, et al., 2021; Bianchi, Tafuri, et al., 2021; Can et al., 2022; Maltezou et al., 2021; Naleway et al., 2022; Ntziora et al., 2022; Redmond et al., 2022). Without exception, these studies focused exclusively on the acute outcomes of COVID-19 infections. Data on long-term consequences of COVID-19 infections in unvaccinated HCPs were notably absent. A recent study found that unvaccinated individuals are at increased risk of developing long-term COVID symptoms, also known as long-COVID (Antonelli et al., 2022). In a large population-based study in the UK, the risk of developing long-COVID was nearly halved among fully vaccinated participants compared to unvaccinated participants (Antonelli et al., 2022). Both Gaber et al. (2021) and Platten et al. (2022) found that nearly half of HCP participants experienced at least one long-COVID symptom following a COVID-19 infection. It is plausible that a substantial number of the unvaccinated HCPs described in this review also proceeded to develop long-COVID symptoms. The dearth of data on this topic must be addressed to better protect unvaccinated HCPs’ physical health and, subsequently, the healthcare workforce from loss of workers to long-term illness (Praschan et al., 2021).

There was a paucity of research on the psychosocial consequences of HCP COVID-19 vaccine hesitancy. As highlighted within Asaoka et al.'s (2022) study on vaccine hesitancy and depression symptoms, a similar study performed among the general population (Palgi et al., 2021) supports the finding of worsening depression scores among vaccine-hesitant HCPs. In Palgi et al.'s (2021) community-based study, COVID-19 vaccine hesitancy was found to be a statistically significant risk factor for both depression and anxiety. However, the link between mental health and COVID-19 vaccine hesitancy appears to be complex, as other researchers have found that people with pre-existing mental illness demonstrate higher levels of COVID-19 vaccine hesitancy than individuals without mental illness (Nguyen et al., 2022; Sekizawa et al., 2022). It is also unclear to what extent the perception of feeling ostracized in the workplace (Eschiti, 2021) or judged by the general public (Dennis et al., 2022) for being vaccine hesitant affects HCPs’ mental health. These findings suggest that the relationship between mental health and vaccine hesitancy must be further clarified, in order to appropriately support HCPs who are both vaccine hesitant and struggling with mental health concerns.

There was a notable lack of data in scholarly peer-reviewed literature on the transmission of COVID-19 from unvaccinated HCPs to patients. A single, non-peer-reviewed outbreak report was identified describing a COVID-19 outbreak in a skilled nursing facility that was precipitated by an unvaccinated staff member (Cavanaugh et al., 2021). A total of 26 residents contracted COVID-19, six residents were hospitalized, and three residents died of COVID-19 (Cavanaugh et al., 2021). Similarly, a study out of South Korea found that nosocomial transmission of COVID-19 was significantly less likely to occur among participants (including patients, caregivers, and HCPs) who had received two doses of a COVID-19 vaccine, compared to those who were partially vaccinated or unvaccinated (Jung et al., 2022). No other articles were encountered during the review process that described these consequences to patients. Similar to challenges encountered in researching the efficacy of COVID-19 immunity passports (Natalia et al., 2023), it is possible that the dearth of data on the efficacy of COVID-19 vaccination in preventing nosocomial transmission of the virus is related to difficulties measuring the effects of multiple simultaneous interventions. For instance, while unvaccinated HCPs have been found to transmit COVID-19 to household members at higher rates than vaccinated HCPs (Shah et al., 2021), this finding cannot be directly translated to healthcare settings in which transmission of COVID-19 may also be reduced by interventions such as superior ventilation strategies, personal protective equipment usage, and policies on attending work with respiratory symptoms. Consequently, detailed documentation on any transmission of COVID-19 from unvaccinated HCPs to patients will be essential for defending the use of COVID-19 vaccine mandates. Influenza “vaccinate-or-mask” policies have previously been overturned when nurses successfully argued that evidence supporting the role of vaccination in reducing influenza transmission from HCPs to patients was inconclusive, and mandatory masking of unvaccinated employees only was not supported by research (De Serres et al., 2017, as cited in Dyer, 2018). High-quality data on COVID-19 transmission between HCPs and patients is therefore needed to guide the development of evidence-based COVID-19 vaccination and infection-control policies.

A noteworthy finding was that HCPs’ COVID-19 vaccine hesitancy was statistically associated with their coworkers’ refusal of the vaccine (Holzmann-Littig et al., 2021). Other studies have conversely demonstrated the favorable role that colleagues play in influencing COVID-19 vaccination decisions. For example, Toth-Manikowski et al. (2022) found that HCPs vaccinated against COVID-19 were more likely than unvaccinated providers to have had their decision positively influenced by colleagues. Likewise, among a sample of critical care HCPs, participants indicated they would have a stronger preference for COVID-19 vaccination if their colleagues were already vaccinated (Huang et al., 2021). These findings suggest that individual HCPs’ COVID-19 vaccine attitudes are both reflective of and contribute toward a broader workplace culture of vaccine acceptance or rejection that healthcare leaders should consider when attempting to address vaccine hesitancy.

Finally, COVID-19 vaccine hesitancy was found to have a negative impact on some healthcare organizations’ staffing levels. While a relatively small number of job losses were described in included articles (Poyiadji et al., 2022; Ritter et al., 2021), it is expected that this consequence could have larger impacts than noted in current scholarly literature. For example, in January 2022, the UK abandoned its plan for mandatory vaccination of all HCPs, as the government anticipated devastating staff shortages (Iacobucci, 2022). Within the NHS, it was expected that 126,000 HCPs would resign rather than accept the COVID-19 vaccine (Kmietowicz, 2021). It will be essential to document staffing-related consequences of HCP vaccine hesitancy in order to determine the safest COVID-19 vaccine policy decisions for HCPs, patients, and healthcare systems alike.

### Strengths and limitations

The strengths of this review include that a reputable review methodology was utilized (Peters et al., 2020). The detailed descriptions of the search strategy and article selection process enhance the rigor of this study and will be conducive to future replicability studies as the research topic continues to evolve. Furthermore, the findings of this review may provide insight into issues to anticipate in future pandemics with novel vaccine rollouts.

Several limitations were also noted during this review. Despite a robust search strategy, relevant articles may have been missed in the search process (Peters et al., 2020). Additionally, quality appraisal was not performed on the included articles, as this does not align with the purpose of a scoping review (Peters et al., 2020). Nevertheless, it was readily apparent that none of the included studies were experimental or quasi-experimental. Given these limitations, causality cannot be ascertained from any of the studies included in this review (Gray, 2021). In many articles, non-vaccination was viewed as a proxy for vaccine hesitancy. It is possible that HCPs medically exempt from vaccination or experiencing challenges with vaccine access were categorized as vaccine hesitant. Additionally, it must be noted that the articles included in this review reflect a short period of the COVID-19 pandemic. It is possible that as the pandemic progressed, some of the documented consequences of HCP COVID-19 vaccine hesitancy—such as job losses related to vaccine mandates—were reversed. Finally, many of the studies in the review took place in high-income countries where COVID-19 vaccines were first made available to the population; therefore, the findings may not be generalizable to low and middle-income countries that experienced inequitable COVID-19 vaccine access (Watson et al., 2022). Despite these limitations, the included sources represent the state of available evidence on the consequences of HCP COVID-19 vaccine hesitancy, and many of the limitations reflect the emerging nature of this research topic.

## Conclusions

The findings of this scoping review suggest that HCP COVID-19 vaccine hesitancy has numerous consequences that negatively affect providers, patients, and the healthcare system. These consequences reflect the complex and intersecting roles of HCPs during a pandemic. For example, HCPs are expected to be leaders in the vaccination effort (Poon et al., 2021) while at high risk of COVID-19 exposure themselves (Gómez-Ochoa et al., 2021). The sum of these consequences demonstrates that COVID-19 vaccine hesitancy among HCPs is an important matter for researchers and healthcare leaders to understand and address.

Accordingly, this scoping review revealed many opportunities for future research. As studies have shown that nurses demonstrate higher levels of COVID-19 vaccine hesitancy than many other HCPS (Browne et al., 2021; Li et al., 2023) and initially suffered the highest rates of COVID-19 infections among HCPs globally (Gómez-Ochoa et al., 2021), researchers should conduct studies that investigate nursing-specific consequences of COVID-19 vaccine hesitancy. Researchers should also use validated instruments to measure HCPs’ levels of COVID-19 vaccine hesitancy, as this would provide more detailed data than the use of binary measures of vaccine acceptance/refusal. Emerging literature suggests that complex interactions exist between COVID-19 vaccine hesitancy and mental health (Asaoka et al., 2022; Nguyen et al., 2022; Sekizawa et al., 2022). This is a topic which requires further research to better understand the supports that HCPs need to feel comfortable accepting vaccination. Finally, research should be performed and repeated in light of the evolving Omicron variants of the COVID-19 virus, to understand the changing implications of HCP vaccine hesitancy over the course of the pandemic.

This review provides implications for current practice. Vaccine-hesitant HCPs, including nurses, should consider that their COVID-19 vaccination views and decisions are not without consequence. Their COVID vaccination decisions extend beyond a personal choice as they potentially risk the health of their colleagues (Redmond et al., 2022) and their workplace's staffing levels (Maltezou et al., 2021; Poyiadji et al., 2022). The findings of this review also reinforce the importance of accurate and considerate vaccine communication among HCPs. While vaccine-hesitant HCPs must be mindful of the COVID-19 vaccination discourse that they convey to patients and colleagues, healthcare leaders and colleagues of vaccine-hesitant HCPs must likewise avoid stigmatizing and belittling hesitant HCPs. Finally, healthcare leaders, researchers, and HCPs should collaboratively develop strategies to prevent and mitigate the consequences of provider vaccine hesitancy to protect HCPs, patients, and healthcare systems alike.

## Supplemental Material

sj-docx-1-cjn-10.1177_08445621241251711 - Supplemental material for Consequences of COVID-19 Vaccine Hesitancy Among Healthcare Providers During the First 10 Months of Vaccine Availability: Scoping Review Supplemental material, sj-docx-1-cjn-10.1177_08445621241251711 for Consequences of COVID-19 Vaccine Hesitancy Among Healthcare Providers During the First 10 Months of Vaccine Availability: Scoping Review by Caitlyn D. Wilpstra, Sherry Morrell, Noeman A. Mirza and Jody L. Ralph in Canadian Journal of Nursing Research
