# Supplementary material for: Consequences of COVID-19 Vaccine Hesitancy Among Healthcare Providers During the First 10 Months of Vaccine Availability: Scoping Review
Source: Can J Nurs Res. 2024 May 2;56(3):204–24. doi: 10.1177/08445621241251711 (PMC11308270; doi:10.1177/08445621241251711)
Supplement: sj-docx-1-cjn-10.1177_08445621241251711 - Supplemental material for Consequences of COVID-19 Vaccine Hesitancy Among Healthcare Providers During the First 10 Months of Vaccine Availability: Scoping Review [file sj-docx-1-cjn-10.1177_08445621241251711.docx]

**Appendix A**

Search Strategy (Example provided for OVID Medline)

| **Set** | **Search Statement** |
| --- | --- |
| #1 | exp health personnel/ or exp nurses/ or exp nursing staff/ or exp pharmacists/ or exp physicians/ |
| #2 | (“healthcare provider*” or “health care provider*” or “healthcare personnel*” or “health care personnel*” or “practitioner*” or “healthcare professional*” or “health care professional*” or “health professional*” or “healthcare worker*” or “health care worker*” or “physician*” or “nurse*” or “pharmacist*”).ti,ab,kw. |
| #3 | 1 or 2 |
| #4 | (vaccin* adj2 (hesitan* or refusal* or denial* or rejection* or attitud* or delay*)).ti,ab,kw. |
| #5 | (immuni* adj2 (hesitan* or refusal* or denial* or rejection* or attitud* or delay*)).ti,ab,kw. |
| #6 | ((mandat* or compuls*) adj2 (vaccin* or immuni*)).ti,ab,kw. |
| #7 | (unvaccinated adj5 (“healthcare provider*” or “health care provider*” or “healthcare personnel*” or “health care personnel*” or “practitioner*” or “healthcare professional*” or “health care professional*” or “health professional*” or “healthcare worker*” or “health care worker*” or “physician*” or “nurse*” or “pharmacist*”)).ti,ab,kw. |
| #8 | 4 or 5 or 6 or 7 |
| #9 | (consequence* or impact* or outbreak* or effect* or recommend* or behavi* or concern* or issue*).ti,ab,kw. |
| #10 | (coronavirus* or “corona virus*” or “COVID*” or “COVID-19*” or “SARS-CoV-2*”).ti,ab,kw. |
| #11 | exp COVID-19 Vaccines/ or exp COVID-19/ |
| #12 | 10 or 11 |
| #13 | 3 and 8 and 9 and 12 |
| #14 | (“healthcare provider*” or “health care provider*” or “healthcare personnel*” or “health care personnel*” or “practitioner*” or “healthcare professional*” or “health care professional*” or “health professional*” or “healthcare worker*” or “health care worker*” or “physician*” or “nurse*” or “pharmacist*”).ti,kw. |
| #15 | (vaccin* adj2 (hesitan* or refusal* or denial* or rejection* or attitud* or delay*)).ti,kw. |
| #16 | (immuni* adj2 (hesitan* or refusal* or denial* or rejection* or attitud* or delay*)).ti,kw. |
| #17 | 15 or 16 |
| #18 | (unvaccinated adj5 (“healthcare provider*” or “health care provider*” or “healthcare personnel*” or “health care personnel*” or “practitioner*” or “healthcare professional*” or “health care professional*” or “health professional*” or “healthcare worker*” or “health care worker*” or “physician*” or “nurse*” or “pharmacist*”)).ti,kw. |
| #19 | 12 and 14 and 17 |
| #20 | 12 and 18 |
| #21 | 13 or 19 or 20 |
